# Understanding Surgical Management and Outcomes in Mitral Valve Endocarditis

**DOI:** 10.3390/jcm14082712

**Published:** 2025-04-15

**Authors:** Elda Dzilic, Samuel Niedermayer, Melchior Burri, Andrea Amabile, Markus Krane, Keti Vitanova

**Affiliations:** 1 Department of Cardiovascular Surgery, Institute Insure, German Heart Center Munich, School of Medicine & Health, Technical University of Munich, 80639 Munich, Germany; dzilic@dhm.mhn.de (E.D.); s.niedermayer@tum.de (S.N.); burri@dhm.mhn.de (M.B.); amabilea@upmc.edu (A.A.); 2 Department of Nephrology, School of Medicine, Klinikum Rechts der Isar, Technical University of Munich, 81675 Munich, Germany; 3 Division of Cardiac Surgery, Department of Cardiothoracic Surgery, University of Pittsburgh Medical Center, Pittsburgh, PA 15213, USA; 4 UPMC Heart and Vascular Institute, University of Pittsburgh Medical Center, Pittsburgh, PA 15213, USA; 5 DZHK (German Center for Cardiovascular Research)—Partner Site Munich Heart Alliance, 80639 Munich, Germany; 6 Division of Cardiac Surgery, Department of Surgery, Yale School of Medicine, New Haven, CT 06510, USA

**Keywords:** mitral valve, endocarditis, mitral valve repair, mitral valve replacement

## Abstract

**Objectives**: Surgical patients with mitral valve endocarditis can be treated with valve reconstruction or valve replacement. Although valve repair should be preferred, the decision between the two options is nuanced. **Methods**: In this single-center, retrospective cohort study, we included all patients who underwent surgery for native mitral valve endocarditis between February 2001 and June 2019. We analyzed the surgical outcomes, survival, and factors leading to valve repair versus replacement. Propensity score matching was performed to minimize treatment assignment bias and improve comparability between the two groups. **Results**: This study included 281 consecutive patients with mitral valve endocarditis, of whom 46 (16.4%) underwent mitral valve repair and 235 (83.6%) underwent mitral valve replacement. The mean follow-up was 5.2 ± 5.1 years. Cases with bileaflet endocarditis (*p* < 0.001), subvalvular apparatus involvement (*p* = 0.008), and abscess formation (*p* = 0.047) were more likely to require valve replacement. The 30-day mortality rate was 12.1% (*n* = 34). Patients who underwent repair had significantly better survival than those who underwent replacement (92.7% ± 4.1% vs. 59.4% ± 3.4% at 5 years; *p* < 0.001), even after propensity score matching (92.6% ± 5.0% vs. 62.4% ± 9.0% at 5 years; *p* = 0.034). **Conclusions**: In patients with mitral valve endocarditis, mitral valve repair had better long-term survival, even after propensity score matching, highlighting the potential benefit of valve preservation techniques.

## 1. Introduction

Surgery for patients diagnosed with mitral valve endocarditis (MVE) remains a significant challenge in the medical community. The 2023 guidelines issued by the European Society of Cardiology (ESC) emphasized the importance of preserving the native valve whenever possible [1]. This approach aligns with the goal of maintaining valve functionality while effectively treating the underlying infection. However, the decision-making process on whether to pursue mitral valve repair (MVr) or replacement (MVR) is complex and influenced by various factors [2].

The choice of surgical approach is influenced by a variety of patient-specific and disease-related factors. In addition, institutional experience and surgical expertise play a critical role in the feasibility and success of MVr. Therefore, real-world data are essential to better understand how decisions are made in daily clinical practice.

Our study aimed to investigate the decision-making process regarding MVr or MVR in our institution. We will examine institutional practices to identify patient-specific and disease-related variables that influence this decision. This investigation aims to provide valuable insights into the considerations that guide treatment decisions in cases of MVE.

## 2. Materials and Methods

We performed a single-center, retrospective cohort study of all consecutive patients who underwent mitral valve (MV) surgery for native MVE at the German Heart Centre Munich between February 2001 and June 2019. Patient data were identified from our internal clinical database. All medical reports, including operative protocols and inpatient and outpatient notes, were reviewed. Data that were collected included demographics, preoperative clinical presentation, microbiological testing, operative data, and postoperative data. Patients with prosthetic valve infective endocarditis were excluded. Referring cardiologists, primary care physicians, as well as patients with prior agreement in writing were contacted to obtain the latest follow-up data.

This study was approved by the ethics committee of the Technical University of Munich (approval reference number: 499/20 S)

Statistical analyses were performed using the Statistical Package for the Social Sciences (SPSS, version 28, IBM Corp, Armonk, NY, USA) and R-Studio for Statistical Computing and Data Science (version 4.2.0, 2022-04-22, RStudio, Boston, MA, USA). Continuous variables were presented as mean (or median) values and standard deviation (or interquartile range) based on the normalcy of distribution; categorical variables were presented as absolute and relative frequencies. The normalcy of distribution was assessed with the Kolmogorov–Smirnov test. An independent sample Student’s *t*-test was used for normally distributed variables. The Mann–Whitney U test was used for non-normally distributed variables. The chi-square test and Fisher’s exact test were used to compare categorical data between the two groups. In all cases, a value of *p* < 0.05 was considered statistically significant. For propensity score matching (PSM), a 1:1 nearest-neighbor matching comparison method without replacement was used. Survival was reported using the Kaplan–Meier method. Differences in outcomes were assessed using the log-rank Mantel–Cox test and cumulative incidence analysis according to the Gray test with a hazard ratio (HR) and 95% confidence interval (CI). Risk factor analysis was performed by fitting a logistic regression model.

## 3. Results

### 3.1. Patient Characteristics

Between 2001 and 2019, a total of 281 patients underwent surgery for MVE, with 95 of them being female (33.8%). The mean age of the patients was 62.0 ± 13.5 years. Out of the total, 65 patients (23.1%) had concomitant aortic valve endocarditis (AVE) and 5 patients (1.8%) had concomitant tricuspid valve endocarditis (TVE). Concomitant surgery was required for non-endocarditis aortic (*n* = 12) and/or tricuspid (*n* = 40) valve disease in 51 patients (18.2%). The percentage of cases indicating non-endocarditis tricuspid valve surgery increased significantly from 9.0% (13 cases) to 20.5% (27 cases) after 2013 (*p* = 0.007).

Before surgery, 80.6% (*n* = 200) of patients presented with severe mitral valve regurgitation, 10.9% (*n* = 27) with moderate, 6.5% (*n* = 16) with mild, and 2.0% (*n* = 5) with no regurgitation. Table 1 summarizes the clinical and demographic data.

The number of patients has increased over the years. From 2001 to 2010, 118 patients (42%) underwent surgery, while 163 patients (58%) were operated on from 2011 to 2019 (*p* = 0.009).

### 3.2. Causative Organism

**Figure 1:**
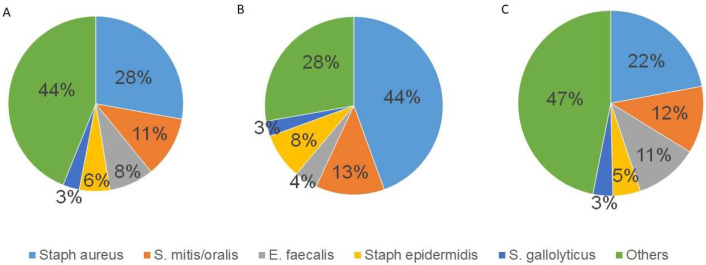
Causative organism: (**A**) spectrum of causative pathogens in all cases; (**B**) spectrum of causative pathogens in females; (**C**) spectrum of causative pathogens in males.

This study identified the causative organism in 77.2% of patients (*n* = 217). *Staphylococcus aureus* (*S. aureus*) was the most common pathogen (*n* = 64; 29.5%), including four cases of methicillin-resistant *S. aureus* (MRSA). Other pathogens are summarized in Figure 1A.

The infection caused by *S. aureus* significantly correlated with abscess formation (r = 0.262, *p* < 0.001) and the presence of vegetation (r = 0.157, *p* = 0.008). Other common organisms, such as *Streptococcus mitis/oralis* (*S. mitis/oralis*; *n* = 26; 11.9%) and *Enterococcus faecalis* (*E. faecalis*; *n* = 19; 8.8%), did not show a correlation with abscess formation (*p* = 0.363 and *p* = 0.453, respectively) or a presence of vegetations (*p* = 0.414 and *p* = 0.759, respectively). Women were significantly more likely than men to develop abscesses (*p* = 0.01). The infection of *S. aureus* did not correlate with the decision to repair or replace the MV (*p* = 0.57) nor with the preoperative degree of MV regurgitation (*p* = 0.59).

The distribution of pathogens is significantly different between men and women. Overall, men have a broader spectrum of pathogens than women (Figure 1B,C). In women, *Staphylococcus aureus* was diagnosed as the causative pathogen in 44% of cases, significantly more than in men (22%, *p* = 0.002).

### 3.3. Operative Data

The surgical procedures were conducted under cardiopulmonary bypass either via median sternotomy (*n* = 253; 90%) or right anterolateral thoracotomy (*n* = 28; 10%). Among the patients who underwent only MV surgery (*n* = 173), 83.8% (*n* = 145) were operated via median sternotomy and 16.2% (*n* = 28) via anterolateral thoracotomy. Those who underwent anterolateral thoracotomy were more often elective cases (*p* < 0.001), had a higher degree of MV regurgitation (*p* = 0.36), and were more likely to receive MV repair (*n* = 17, 60.7%).

During intraoperative MV assessment, vegetations were found on one leaflet in 38.8% (*n* = 95) of the cases, on both leaflets in 53.4% (*n* = 150) of the cases, on the subvalvular apparatus in 26.7% (*n* = 75) of the cases, and an abscess was present in 10.3% (*n* = 29) of the cases.

MV repair was achieved in 46 patients (16.4%) using annuloplasty devices (*n* = 44), leaflet repair techniques (*n* = 39), and artificial chordae replacement (*n* = 22). Leaflet repair was performed using patch plasty with autologous pericardium in 11 cases, CardioCel^®^ in 2 cases, and Gore-Tex^®^ in 1 case. Leaflet resection was performed in 23 cases, and direct suture of a lesion was performed in 7 cases. In the 22 patients who underwent artificial chordae replacement, an average of 2.55 chordae were used. MV replacement was performed in 195 patients (69.4%) with a bioprosthetic valve and in 40 patients (14.2%) with a mechanical valve.

A total of 217 (77.2%) procedures were performed as urgent or emergent. In these cases, MV replacement (*n* = 192) was significantly more common than MV repair (*n* = 25) (*p* < 0.001). The mean bypass time was 133.6 ± 53.8 min, with a mean cross-clamp time of 94.6 ± 39.7 min. There were no significant differences in the mean bypass time (*p* = 0.634) or mean cross-clamp time (*p* = 0.892) between patients who received MV repair and those who received MV replacement. There was no statistically significant difference between MVr and MVR in patients with concomitant procedures (AV or TV surgery) (*p* = 0.121).

Cases with bileaflet MV endocarditis (*p* < 0.001), subvalvular apparatus involvement (*p* = 0.008), and abscess formation (*p* = 0.047) required MV replacement more frequently.

The mean age of the patients undergoing MV repair was significantly lower (52.8 ± 13.5 years) than those undergoing MV replacement (63.8 ± 12.8 years) (*p* < 0.001). MV repair was performed more frequently in men (*n* = 37, 80.4%) than in women (*n* = 9, 19.6%) (*p* = 0.026). A slight negative correlation was observed between the preoperative degree of MV regurgitation and MV replacement (r = −0.138, *p* = 0.030). Additional surgical procedure details are provided in Table 2.

### 3.4. Postoperative Course

The patients had a mean ICU stay of 8.3 ± 10.5 days (median 4). There was a significant difference in the length of stay between patients who underwent MV repair (3.9 ± 3.5 days) and those who underwent MV replacement (9.1 ± 11.2 days) (*p* < 0.001). Postoperative hemodialysis was required for 61 patients (21.7%), and 15 patients (5.3%) experienced postoperative neurological symptoms, including stroke (*n* = 12, 4.3%), TIA (*n* = 1; 0.4%), and seizures (*n* = 2; 0.7%). This study found that patients who underwent MV replacement were more likely to require postoperative dialysis (25%) compared to those who underwent MV repair (4.3%) (*p* = 0.002). However, there was no significant difference in the incidence of stroke between the two groups (*p* = 0.117). The mean hospital stay was 15.2 ± 12.3 days (median 12), with a significant difference between patients who underwent MV repair (11.4 ± 5.6 days) and those who underwent MV replacement (16.0 ± 13.1 days) (*p* < 0.001) (Table 3).

### 3.5. Survival

**Figure 2:**
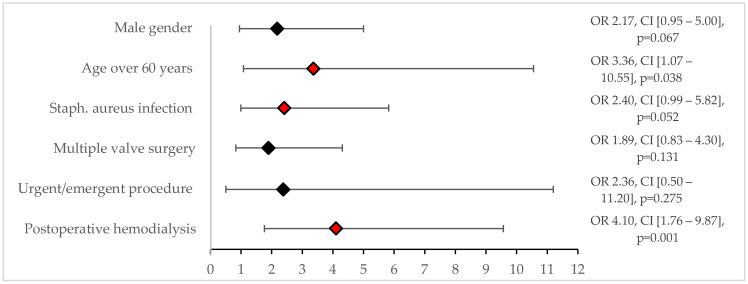
Predictors for 30-day mortality. Black diamonds indicate no statistical significance. Red diamonds indicate statistical significance.

The mean follow-up period was 5.2 ± 5.1 years. The 30-day mortality rate was 12.1% (*n* = 34). Risk factors for 30-day mortality were identified through univariate analysis, including an *S. aureus* infection (*p* < 0.001), postoperative need for dialysis (*p* < 0.001), age over 60 years (*p* = 0.002), male sex (*p* = 0.005), multiple valve surgery (*p* = 0.028), and urgent/emergent surgery (*p* = 0.046). Multivariate logistic regression using significant factors from the univariate analysis revealed that an *S. aureus* infection (OR 2.4, CI [0.9–5.8]), age over 60 years (OR 3.4, CI [1.1–10.5]), and the need for postoperative dialysis (OR 4.1, CI [1.7–9.6]) were significant predictors of 30-day mortality (Figure 2).

**Figure 3:**
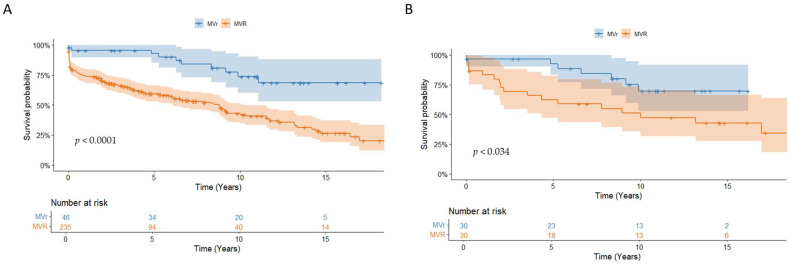
Survival: (**A**) overall survival of all patients; (**B**) overall survival after propensity score matching.

**Figure 4:**
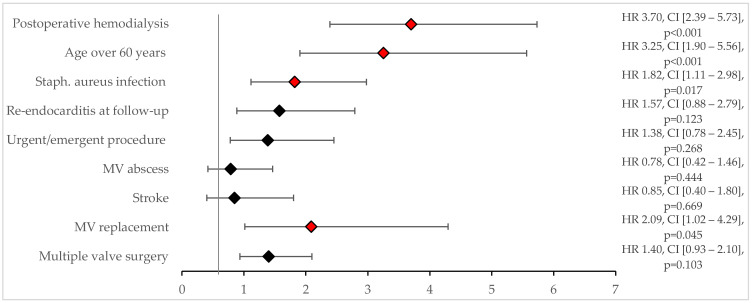
Predictors for overall mortality. Black diamonds indicate no statistical significance. Red diamonds indicate statistical significance.

This study found that the overall survival rates were 77.5% ± 2.5% at 1 year, 64.6 ± 3.0% at 5 years, and 47.8 ± 3.7% at 10 years. Patients who underwent MV repair had significantly better survival rates compared to those who underwent MV replacement (92.7% ± 4.1% vs. 59.4% ± 3.4% at 5 years; *p* < 0.001) (Figure 3A). The Cox regression analysis revealed that a recurrence of endocarditis (HR 2.8, CI [1.5–5.2]; *p* = 0.001), median sternotomy (HR 5.1, CI [1.2–21.7]; *p* = 0.026), and MV replacement (HR 2.2, CI [1.0–4.7], *p* = 0.041) were significant predictors of all-cause mortality (Figure 4).

As patients receiving MVR tended to be older and had more extensive valve damage, PSM was performed and yielded 30 pairs. The baseline characteristics after PSM were comparable for important variables such as sex, age, and degree of endocarditis, as shown in Table 4. After PSM, survival was still significantly better in patients who underwent MVr compared to those who underwent MVR (92.6% ± 5.0% vs. 62.4% ± 9.0% at 5 years; *p* = 0.034) (Figure 3B).

### 3.6. Reinfection

Twenty-five patients (8.9%) experienced recurrent endocarditis after an average of 2.1 ± 3.0 years, ranging from 11 to 3914 days (median 284 days). The cumulative incidence of reinfection at 10 years was 8.4% ± 1.9%.

Of these patients, 19 (76%) experienced reinfection of the prosthetic valve (14 mitral valves and three aortic valves), while 6 patients (24%) experienced reinfection of the reconstructed mitral valve. There was no significant difference in the reinfection rate between mitral valve repair and replacement (*p* = 0.320). The univariate analysis revealed that multiple valve surgery was a predictive factor for reinfection (*p* = 0.025). The presence of *S. aureus* infection (*p* = 0.364), vegetation (*p* = 0.110), abscess (*p* = 0.828), involvement of the subvalvular apparatus (*p* = 0.772), or urgency of surgery (*p* = 0.077) did not have a significant effect on the recurrence of infection. Seventeen patients with recurrent endocarditis required reoperation.

## 4. Discussion

This retrospective, single-center study reviews the surgical landscape of MVE over nearly two decades of experience and provides key insights into surgical decision-making for the indication and successful performance of mitral valve repair in MVE patients: (1) The decision for MVR was often based on extensive valve destruction and higher surgical urgency. (2) MVr was more often performed in younger male patients. (3) Female patients had a different pathogen spectrum with a higher rate of *S. aureus* infections compared to male patients. (4) MVr for MVE resulted in better long-term survival and was not associated with higher reinfection rates. (5) *S. aureus* infection was associated with more invasive disease and higher 30-day and overall mortality rates.

### 4.1. Repair or Replace

Our data and other studies indicate a significant increase in cardiac surgery for MVE over the past two decades [3,4]. This increase is partly due to an aging population with a growing prevalence of implantable cardiac devices, a known risk factor for endocarditis [5]. Additionally, advances in cardiovascular medicine now allow surgery in patients who were once considered inoperable despite the increasing complexity of their disease. Our manuscript casts light on how the growing number of complex patients should be managed. According to the 2023 ESC guidelines, a careful approach is recommended for MVE surgery, considering repair or replacement [1]. While MVr is generally preferred, the decision should be based on several factors that are not yet fully defined. Real-world data show that the repair rates vary greatly, ranging from 10.7% to 80.7% [2,6,7].

In our cohort, 16.4% of patients underwent MVr. Upon closer examination of the MVr group, several factors stand out:

(1) Patients in the MVr group were younger than those who received MVR, similar to previous data [6]. This suggests a deliberate focus on valve preservation in this population. The choice between biological and mechanical valve replacement is a complex dilemma in this particular age group [8,9]. Although mechanical valves offer superior durability, they require lifelong anticoagulation therapy, which can pose significant challenges and risks. In contrast, biological valves do not require long-term anticoagulation but may have a shorter lifespan, potentially requiring repeat surgery in the future. The decision-making process must weigh the trade-offs between valve durability and the avoidance of anticoagulation-related complications. Factors such as the patient’s lifestyle, preferences, and long-term prognosis should be considered. MVr offers patients a durable solution, avoiding this dilemma.
(2) The decision to choose MVr was based on the severity and extent of the endocarditis rather than the number of concomitant procedures performed. Surgeons often preferred MVR when there was extensive valve destruction, as indicated by the presence of bileaflet vegetation, involvement of the subvalvular apparatus, and abscess formation. When the valve’s structural integrity is severely compromised, repair may not be a feasible or durable solution to address the underlying issue. Concerns that valve preservation leads to an increased rate of reinfection are unfounded [6,10]. There is no significant difference in reinfection rates between MVr and MVR patients. Moreover, several studies have shown a lower reinfection rate, especially in the long term [10,11,12]. This may be due to the reduced amount of prosthetic material in MVr compared to MVR.
(3) The spectrum of pathogens differed between male and female patients. Female patients had more frequent *S. aureus* infections, which led to more extensive valve damage, as evidenced by abscess formation. The more invasive disease meant that female patients were less likely to undergo MVr compared to male patients. Similarly, Defauw et al. showed, in their cohort, that male patients underwent MV repair more often than females [10]. The group could not provide reasons for their observation. However, Bansal et al. showed that female patients were generally less likely to undergo cardiac surgery when diagnosed with infective endocarditis [13]. The exact reason for this bias remains unclear. However, this gender disparity raises important questions about the potential differences in symptom presentation between men and women with mitral valve endocarditis. A delayed diagnosis may lead to more advanced disease progression, which may impact the feasibility and efficacy of MVr. As a result, surgeons may prefer MVR as a more definitive solution, especially in cases where the valve damage is extensive. One approach to improving mitral valve repair rates in female patients may be timely surgical intervention in cases of *S. aureus*-induced endocarditis. The aggressive nature of *S. aureus* has been well documented in this and other studies, with infections often leading to abscess formation and septic shock [1]. Given these characteristics, special vigilance is warranted in female patients, especially when *S. aureus* is microbiologically confirmed.
(4) Patients receiving MVR were more likely to be urgent or emergent cases. The emergent nature of the condition often means that patients present with a worse clinical status. As a result, surgeons may favor the potentially faster option of valve replacement over attempts at valve reconstruction [10,14]. Moreover, emergency cases may be handled by surgeons who may not have extensive experience with MVr. Consequently, they may lean toward valve replacement, which is a more straightforward procedure in such circumstances.

### 4.2. Survival

Survival rates after MVr and MVR for MVE vary widely, ranging from a 7.8% long-term mortality after repair to 40.5% mortality after replacement [2]. However, comparing patients undergoing mitral valve reconstruction to those undergoing replacement for the treatment of MVE presents inherent challenges due to the different characteristics of the patient populations in each group [15]. As previously outlined, patients undergoing reconstruction tend to be younger, present with less severe forms of endocarditis, and are less likely to require urgent intervention. This difference is also reflected in the postoperative course. Patients with reconstructed valves require less postoperative renal replacement therapy and have shorter stays in intensive care and the hospital. It is, therefore, not surprising that reconstruction leads to better survival outcomes than replacement, as has been shown previously [6,16,17]. To make the groups more comparable, we performed PSM. This confirmed the survival advantage after valve reconstruction. The observed survival benefit may be due to the preservation of ventricular anatomy after valve repair [18]. However, Toyoda et al. could not confirm the better outcome after PSM in patients undergoing MV surgery for MVE [6].

### 4.3. Risk Factors

Although many risk factors for mortality are not directly modifiable (e.g., age, need for dialysis), indirect intervention is still possible. Our research, along with other evidence, emphasizes the association between *S. aureus* infection and an increased likelihood of abscess formation [19]. The presence of abscesses significantly worsens disease progression, underscoring the importance of early detection and intervention [20]. When *S. aureus* infection is identified, it is necessary to exercise heightened vigilance through more frequent echocardiographic monitoring to rapidly detect disease progression. In particular, pulsed wave tissue Doppler imaging (PW-TDI) is emerging as a valuable modality for the assessment of intracardiac masses, such as valvular vegetations. By enabling the assessment of vegetation mobility, PW-TDI provides an important surrogate marker for the likelihood of embolic events [21,22]. This proactive approach facilitates timely decision-making regarding the need for surgery, thereby reducing potential complications [23]. By implementing these measures, the chances of patients being eligible for valve repair are increased, providing a promising approach to improving outcomes in the treatment of mitral valve endocarditis.

As described previously, the avoidance of prosthetic material by MVr leads to lower reinfection rates long term [10,11,12]. This is especially important in patients requiring multiple valve surgery. Our data show that multiple valve surgery is a risk factor for reinfection. By reducing the amount of prosthetic material, reinfection rates may be decreased.

## 5. Conclusions

The decision between repair and replacement was influenced by the severity and extent of endocarditis, with MVR often favored in cases of extensive valve destruction. MVr was performed in younger male patients with less valve destruction. Gender differences in surgical treatment highlight a different spectrum of pathogens and potential differences in symptom presentation between men and women and underscore the need for early diagnosis and intervention. Mitral valve repair in patients with MVE resulted in better short- and long-term outcomes than MVR, even in a matched group comparison.

## 6. Limitations

The limitations of this study are consistent with the design of a retrospective analysis study. This study used data from a single center. Selection bias and confounding bias cannot be ruled out. The differences in patient characteristics between the two cohorts make it difficult to compare the two groups. Although PSM was performed, other differences between the groups may have been missed.

## Figures and Tables

**Table 1:** Baseline characteristics before PSM.

	All	MV Repair	MV Replacement	*p*-Value
Age (years)	62.0 ± 13.5	52.8 ± 13.5	63.8 ± 12.8	<0.001
Female	95	9 (9.5)	86 (90.5)	<0.001
Male	186	37 (19.9)	149 (80.1)	<0.001
Diabetes mellitus	47 (16.7)	6 (13.0)	41 (17.4)	0.7
Hypertension	123 (43.8)	14 (30.4)	109 (46.4)	0.052
AV endocarditis	65 (23.1)	7 (15.2)	58 (24.7)	0.2
TV endocarditis	5 (1.8)	0 (0.0)	5 (2.1)	1.0
Preoperative cerebral embolization	49 (17.4)	9 (19.6)	40 (17.0)	0.7

Data are presented as *n* (%) or means ± standard deviations. AV: aortic valve; TV: tricuspid valve.

**Table 2:** Procedural information.

	All	MV Repair	MV Replacement	*p*-Value
Urgent/Emergent	217 (77.2)	25 (54.3)	192 (81.7)	<0.001
Cardiopulmonary bypass time (mins)	133.6 ± 53.8	130.1 ± 47.1	134.3 ± 55.1	0.634
Cross-clamp time (mins)	94.6 ± 39.7	95.4 ± 35.1	94.5 ± 40.6	0.892
Monoleaflet MV endocarditis	95 (33.8)	17 (37.0)	78 (33.2)	0.6
Bileaflet MV endocarditis	150 (53.4)	14 (30.4)	136 (57.9)	0.001
Involvement of subvalvular Apparatus of MV	75 (26.7)	5 (10.9)	70 (29.8)	0.006
Abscess MV	29 (10.3)	1 (2.2)	28 (11.9)	0.046
AV surgery	77 (27.4)	8 (17.4)	69 (29.4)	0.11
TV surgery	45 (16.0)	6 (13.0)	39 (16.6)	0.7

Data are presented as *n* (%) or means ± standard deviations. MV: mitral valve; AV: aortic valve; TV: tricuspid valve.

**Table 3:** Postoperative information.

	MV Repair	MV Replacement	*p*-Value
ICU stay (days)	3.9 ± 3.5	9.1 ± 11.2	*p* < 0.001
Hospital stay (days)	11.4 ± 5.6	16.0 ± 13.1	*p* < 0.001
Hemodialysis	2 (4.3%)	59 (25.1%)	*p* < 0.001
Postoperative stroke	0 (0.0)	12 (5.1%)	*p* = 0.117

Data are presented as *n* (%) or means ± standard deviations. ICU: intensive care unit.

**Table 4:** Patients’ characteristics after propensity score matching.

	All Matched Patients	MV Repair	MV Replacement	*p*-Value
Age (years)	56.1 ± 11.5	56.3 ± 11.8	55.9 ± 11.4	0.91
Female	12	6 (50)	6 (50)	1.0
Male	46	24 (50)	24 (50)	1.0
Diabetes mellitus	8 (13.3)	5 (62.5)	3 (37.5)	0.81
Preoperative cerebral embolization	7 (11.7)	6 (85.7)	1 (14.3)	0.04
AV endocarditis	8 (13.3)	4 (50)	4 (50)	1.0
Urgent/Emergent	37(61.7)	17 (46.0)	20 (54.0)	0.29
Cardiopulmonary bypass time (mins)	125.3 ± 41.3	126.1 ± 33.5	124.5 ± 48.2	0.88
Cross-clamp time (mins)	92.0 ± 30.1	92.4 ± 24.5	91.6 ± 35.0	0.91
Vegetation	50 (83.3)	25 (50)	25 (50)	1.0
Abscess MV	0	0 (0.0)	0 (0.0)	

Data are presented as *n* (%) or means ± standard deviations.

## Data Availability

The data presented in this study are available on request from the corresponding author.

## Abbreviations

The following abbreviations are used in this manuscript:

AV	Aortic valve
AVE	Aortic valve endocarditis
CI	Confidence interval
ESC	European Society of Cardiology
HR	Hazard ratio
ICU	Intensive care unit
MRSA	Methicillin-resistant *S. aureus*
MV	Mitral valve
MVE	Mitral valve endocarditis
MVr	Mitral valve repair
MVR	Mitral valve replacement
OR	Odds ratio
PSM	Propensity score matching
PW-TDI	Pulsed wave tissue Doppler imaging
TIA	Transient ischemic attack
TV	Tricuspid valve
TVE	Tricuspid valve endocarditis
